# Associations of Perceived Stress and Psychological Resilience With Cognition and a Modifiable Dementia Risk Score in Middle-Aged Adults

**DOI:** 10.1093/geronb/gbad131

**Published:** 2023-09-18

**Authors:** Katherine H Franks, Lisa Bransby, Lachlan Cribb, Rachel Buckley, Nawaf Yassi, Trevor T -J Chong, Michael M Saling, Yen Ying Lim, Matthew P Pase

**Affiliations:** Turner Institute for Brain and Mental Health, School of Psychological Sciences, Monash University, Clayton, Victoria, Australia; Turner Institute for Brain and Mental Health, School of Psychological Sciences, Monash University, Clayton, Victoria, Australia; Turner Institute for Brain and Mental Health, School of Psychological Sciences, Monash University, Clayton, Victoria, Australia; Department of Neurology, Massachusetts General Hospital and Harvard Medical School, Boston, Massachusetts, USA; Department of Neurology, Center for Alzheimer Research and Treatment, Brigham and Women’s Hospital, Boston, Massachusetts, USA; Department of Medicine and Neurology, Melbourne Brain Centre at the Royal Melbourne Hospital, University of Melbourne, Parkville, Victoria, Australia; Population Health and Immunity Division, The Walter and Eliza Hall Institute of Medical Research, Parkville, Victoria, Australia; Turner Institute for Brain and Mental Health, School of Psychological Sciences, Monash University, Clayton, Victoria, Australia; Melbourne School of Psychological Sciences, University of Melbourne, Parkville, Victoria, Australia; Florey Institute of Neuroscience and Mental Health, University of Melbourne, Parkville, Victoria, Australia; Turner Institute for Brain and Mental Health, School of Psychological Sciences, Monash University, Clayton, Victoria, Australia; Turner Institute for Brain and Mental Health, School of Psychological Sciences, Monash University, Clayton, Victoria, Australia; Harvard T.H. Chan School of Public Health, Harvard University, Boston, Massachusetts, USA

**Keywords:** CAIDE, CD-RISC, Cognitive functioning, Cross-sectional, PSS

## Abstract

**Objectives:**

Psychological stress has been proposed as a risk factor for cognitive impairment and dementia. However, it remains unclear how an individual’s stress-coping ability (i.e., psychological resilience) is related to cognition. This cross-sectional study investigated whether perceived stress and psychological resilience were associated with cognition and a modifiable dementia risk score in a large community-based sample of cognitively normal adults. The moderating effect of psychological resilience was also examined.

**Methods:**

Participants (mean age = 57 ± 7 years) enrolled in the web-based Healthy Brain Project completed the Perceived Stress Scale and the Connor–Davidson Resilience Scale. Domains of attention and working memory were assessed using the Cogstate Brief Battery (*n* = 1,709), and associative memory was assessed using the Cambridge Neuropsychological Test Automated Battery (*n* = 1,522). Dementia risk was estimated for 1,913 participants using a modified version of the Cardiovascular Risk Factors, Aging, and Incidence of Dementia dementia risk score, calculated using only readily modifiable dementia risk factors.

**Results:**

In separate linear regression analyses adjusted for age, sex, education, and race, greater levels of perceived stress and lower levels of psychological resilience were associated with poorer performance across all cognitive domains, as well as a higher modifiable dementia risk score. Psychological resilience did not moderate the effect of perceived stress on cognition or the dementia risk score.

**Discussion:**

Higher perceived stress and lower resilience were associated with poorer cognition and a greater burden of modifiable dementia risk factors. Intervention studies are required to determine if lowering stress and building resilience can mitigate cognitive deficits and reduce dementia risk.

Epidemiological estimates suggest that up to 40% of dementia cases can be attributed to 12 modifiable risk factors, including obesity, excessive alcohol intake, hypertension, less education, traumatic brain injury, hearing loss, diabetes, air pollution, physical inactivity, social isolation, depression, and smoking (Livingston et al., 2020). In the absence of readily available and effective disease-modifying treatments for dementia, targeting such modifiable risk factors is a promising means to reduce dementia prevalence. However, not all of the 12 identified risk factors are necessarily readily modifiable; for example, although one can attempt to prevent a traumatic brain injury, once sustained, this is no longer modifiable. Furthermore, reducing exposure to air pollution may be out of an individual’s control. As such, elucidating additional risk factors for cognitive impairment could suggest new avenues for dementia prevention. Emerging evidence suggests that psychological stress may be a risk factor for the development of cognitive impairment and dementia in late life (Franks et al., 2021, 2022), and thus could be a potential target for interventions to reduce dementia risk.

Psychological stress occurs when an individual perceives demands as stressful and as exceeding their coping resources (Lazarus & Folkman, 1984). It has been postulated that dysregulation of the hypothalamic-pituitary-adrenal (HPA) axis and subsequent elevated levels of glucocorticoids may underlie the relationship between chronic psychological stress and cognitive impairment (Canet et al., 2019; Ouanes & Popp, 2019). If sustained, chronically elevated glucocorticoid levels can contribute to structural changes, particularly in the hippocampus and prefrontal cortex, which might, in turn, have negative consequences for cognition (Canet et al., 2019; Ouanes & Popp, 2019). Alternatively, psychological stress may contribute to cognitive impairment and dementia risk by way of its impact on adverse coping behaviors and cardiovascular health. Stress has been linked to physical inactivity (Rod et al., 2009), smoking (Rod et al., 2009), and hypertension (Liu et al., 2017), which are well-established dementia risk factors (Livingston et al., 2020). Indeed, midlife cardiovascular factors have been found to predict dementia risk in late life and are incorporated in the well-validated Cardiovascular Risk Factors, Aging, and Incidence of Dementia (CAIDE) dementia risk score (Kivipelto et al., 2006).

Importantly, not every individual who experiences stress will develop cognitive impairment or dementia. Psychological resilience is one factor that might help to protect against the negative effects of stress. The conceptualization of psychological resilience varies, but it is typically viewed as a *trait* (i.e., a broadly stable personality trait) or *process* (i.e., a response to adversity or stress that is dynamic; MacLeod et al., 2016; Windle, 2011). More broadly, it captures personal characteristics that allow an individual to cope with stress or adversity (Connor & Davidson, 2003). Greater resilience has been associated with more positive outcomes in later life, such as increased quality of life and lower levels of depression (MacLeod et al., 2016). However, it is unclear whether this extends to cognition and dementia risk. Given that constructs related to resilience (i.e., psychological well-being) are positively associated with cognition, it is plausible that a relationship may also exist between psychological resilience and cognition (Llewellyn et al., 2008). Although some studies have found a relationship between resilience and subjective (but not objective) cognition (Arola et al., 2021; Lamond et al., 2008), others have supported the link between psychological resilience and better objective cognition (Deng et al., 2018; Jung et al., 2021; Ma et al., 2022; McDaniel et al., 2022). However, many of these studies focused on narrowly defined samples (e.g., military veterans, solely women or men, and young adults). More research is therefore needed to determine whether psychological resilience is associated with cognition and dementia risk, especially in community-dwelling adults.

Psychological resilience may be associated with cognition and dementia risk through multiple different pathways. One possibility is that greater resilience is associated with better coping behaviors, which are themselves linked to better cognitive function (Ma et al., 2022). A second possibility is that psychological resilience protects against the negative effects of chronic stress. Indeed, research has proposed that psychological resilience might modify the association between HPA axis dysregulation and psychological health in older adults (Gaffey et al., 2016). As such, it is plausible that psychological resilience could also modify the association of psychological stress with cognition and dementia risk. This relationship is important to investigate because boosting resilience could be a potential intervention strategy to minimize the impact of perceived stress on cognitive impairment. Indeed, a recent meta-analysis has shown that psychological resilience is modifiable through psychological interventions (Joyce et al., 2018). However, it remains unknown if psychological resilience can buffer against the potential negative effects of psychological stress on cognition and dementia risk.

Exploring relationships between perceived stress, cognition, and dementia risk, as well as factors that protect against the negative effects of stress (e.g., psychological resilience), is important for the future development of dementia prevention guidelines. Furthermore, it is important to investigate these relationships in midlife, prior to the onset of cognitive impairment or substantial neurodegenerative changes, as dementia prevention initiatives may be most effective at this time. Therefore, we investigated the cross-sectional relationships of perceived stress and psychological resilience with both cognition and a modified dementia risk score in a large web-based sample of middle-aged adults, who were enriched for a family history of dementia. Specifically, our first aim was to investigate the separate associations of perceived stress and psychological resilience with cognition. We hypothesized that greater levels of perceived stress and lower levels of resilience would both be associated with poorer cognition. Our second aim was to investigate the separate associations of perceived stress and psychological resilience with a modified dementia risk score. We hypothesized that greater levels of perceived stress and lower levels of psychological resilience would be associated with a higher burden of modifiable dementia risk factors, given the known negative effects of stress on cardiovascular health (Steptoe & Kivimäki, 2012) and unhealthy coping behaviors (Rod et al., 2009). Our third aim was to investigate whether any associations of perceived stress with cognition or dementia risk scores were moderated by psychological resilience.

## Method

### Participants

The current study included participants enrolled in the Healthy Brain Project (HBP), an online prospective community-based study of adults aged between 40 and 70 years at enrollment. The study has been enriched to include a high percentage of participants with a family history of dementia. The inclusion and exclusion criteria of the HBP have been described elsewhere (Lim et al., 2019). In brief, participants are recruited from across Australia, with every state and territory represented, and with 25% of participants reporting postcodes from regional or remote Australia. Exclusion criteria include a self-reported history of severe traumatic brain injury, diagnosis of dementia including Alzheimer’s disease (AD), diagnosis of Parkinson’s disease, use of medications for the symptomatic treatment of AD, diagnosis of a psychiatric disorder within the past year, uncontrolled major depression, or a history of substance abuse or dependence within the past two years.

**Figure 1.**
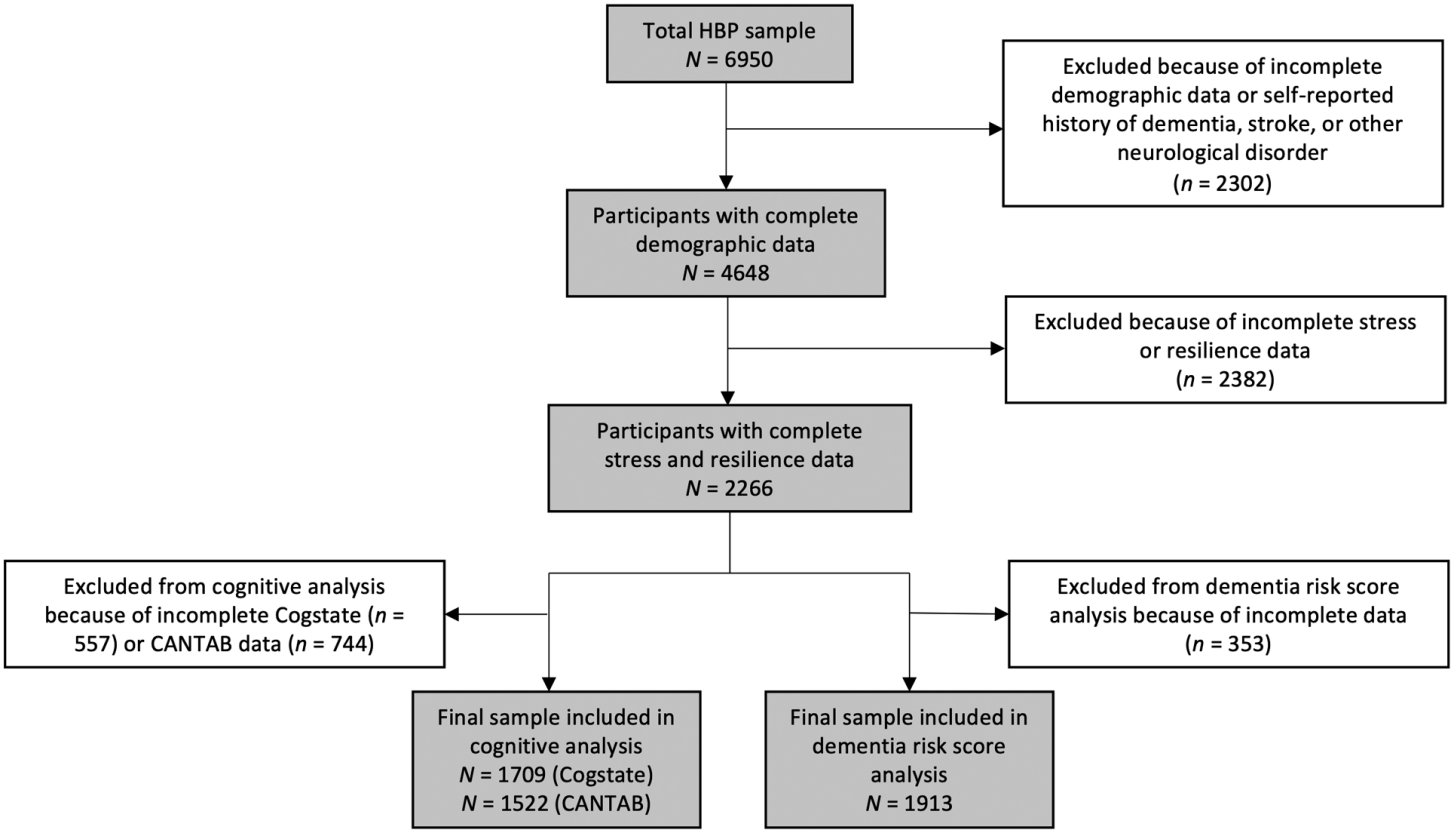
Participant flowchart. CANTAB = Cambridge neuropsychological test automated battery; HBP = Healthy Brain Project.

The HBP was launched on February 1, 2017, and recruitment and assessment of HBP participants are ongoing. For this cross-sectional study, we report baseline data collected up to and including December 6, 2020. Participants were excluded if they had a self-reported history of dementia, stroke or any other neurological disorder, or had incomplete demographic data. In total, 2,266 participants completed measures of psychological stress and resilience. Of these, 1,709 participants completed the Cogstate Brief Battery and 1,522 completed the Cambridge Neuropsychological Test Automated Battery (CANTAB). Additionally, 1,913 participants had available data to calculate the modified dementia risk score (see Figure 1). All participants provided informed consent and study approval was obtained from the Monash University Human Research Ethics Committee.

### Measures

Participants used the online HBP platform (healthybrainproject.org.au) to complete all measures and questionnaires. Participants had a 6-month window to complete all assessments but were encouraged to complete all assessments within 1 month. They could complete the questionnaires across multiple sessions but were required to complete each cognitive measure (i.e., the Cogstate Brief Battery and the CANTAB) in one session.

### Perceived Stress

Perceived stress was measured using the 10-item Perceived Stress Scale (PSS), which assesses the extent to which individuals have perceived situations as stressful within the past month (Cohen et al., 1983). The 10-item PSS is a reliable and valid scale of perceived stress (Cohen & Williamson, 1988; Ezzati et al., 2014). Participants rated each item on a 5-point Likert scale, ranging from “never” to “very often.” Items included questions such as “how often have you felt nervous and stressed?” and “how often have you felt difficulties were piling up so high that you could not overcome them?.” Reverse items were recorded (e.g., “how often have you felt that you were on top of things?”). The outcome was the total score, with higher scores indicating greater levels of perceived stress (range 0–40). The PSS had a high level of internal consistency in our sample (Cronbach’s alpha = 0.90).

Participants completed the PSS a median of 0.3 weeks prior to the Cogstate Brief Battery (IQR = 8.7 weeks), and 1.4 weeks prior to the CANTAB (IQR = 4.5).

### Psychological Resilience

The 10-item version of the Connor–Davidson Resilience Scale (CD-RISC) was used as a measure of trait resilience and stress-coping ability (Campbell-Sills & Stein, 2007; Connor & Davidson, 2003). The 10-item version of the CD-RISC has excellent psychometric properties and is comparable to the original 25-item scale (Campbell-Sills & Stein, 2007). For each item, participants rated how much each statement applied to them over the past month using a 5-point Likert scale, ranging from “not true at all” to “true nearly all of the time.” Items included statements such as “I think of myself as a strong person when dealing with life’s challenges and difficulties” and “having to cope with stress can make me stronger.” Scores were totaled, with higher scores reflecting greater levels of resilience (range 0–40). The CD-RISC had a high level of internal consistency in our sample (Cronbach’s alpha = 0.91).

The CD-RISC was added to the online HBP platform on December 10, 2019. As a result, participants completed the baseline CD-RISC with a median of 2.0 years (IQR = 1.9) following the Cogstate Brief Battery, and 1.9 years (IQR = 1.7) following the CANTAB (a sensitivity analysis taking into account this delay did not significantly change our findings—see *Results*). The CD-RISC has acceptable test–retest reliability, suggesting relative stability over time (Connor & Davidson, 2003).

### Cognition

Participants completed the Cogstate Brief Battery, which has been optimized for remote, unsupervised assessment (Mackin et al., 2018; Perin et al., 2020). The Cogstate Brief Battery comprises four subtests that use playing card stimuli: Detection (DET), Identification (IDN), One Card Learning (OCL), and One-Back (OBK). These subtests have been comprehensively described elsewhere (Lim et al., 2012). Briefly, DET is a simple reaction time task measuring psychomotor function and IDN is a choice reaction time paradigm measuring visual attention. The main outcome measure of these subtests was reaction time (ms), which was normalized using a log10 transformation. OCL is a test of continuous visual recognition task measuring visual learning and OBK is an *n*-back paradigm measuring working memory. The main outcome measure of these subtests was accuracy (proportion correct), normalized using an arcsine square-root transformation. For each subtest, the mean and standard deviation of the sample was calculated. These were used to standardize raw scores to *z* scores for each participant. The *z* scores for DET and IDN were reversed so that lower values reflected poorer performance (slower response times). An attention/psychomotor function composite score (attention composite) was computed by averaging the *z* scores for DET and IDN subtests and a learning/working memory composite score (working memory composite) was computed by averaging the *z* scores for OCL and OBK subtests, as reported previously (Maruff et al., 2013). The Cogstate Brief Battery has been shown to be sensitive to cognitive impairment related to mild cognitive impairment (MCI) and AD (Lim et al., 2012; Maruff et al., 2013).

Participants also completed the Paired Associates Learning (PAL) subtest from the CANTAB, which has been validated for unsupervised cognitive assessment (Backx et al., 2020). This subtest is a measure of arbitrary associative memory. It involves a nonverbal association paradigm that arbitrarily pairs objects and locations. Outcome measures include total errors adjusted and the first attempt memory score. Given the abnormal multimodal distribution of the total errors adjusted in the current sample, we used the first attempt memory score, which is the frequency that participants responded correctly on the first attempt across all trials, as previously described (Abbott et al., 2019; Backx et al., 2020). The mean and standard deviation of the sample were calculated and used to standardize raw scores to *z* scores for each participant. Higher scores indicated better performance. The PAL subtest has been shown to be sensitive to cognitive changes in MCI and AD dementia (De Jager et al., 2005; Fowler et al., 2002).

### Dementia Risk Score

A modified version of the well-validated CAIDE dementia risk score based on only those risk factors that are readily modifiable was estimated for each participant, as previously reported (Pase et al., 2022). Risk factors were calculated using self-reported data on hypertension, cholesterol, body mass index, and physical activity (see Supplementary Material). The score from each factor was summed to calculate the total dementia risk score attributable to readily modifiable risk factors. We included the modified CAIDE (as opposed to the original CAIDE score, which includes chronological age, biological sex, and years of education) because we were interested in estimating the portion of dementia risk that is readily modifiable. Furthermore, we wanted to capture the dementia risk factors that could theoretically be influenced by perceived stress and psychological resilience. Chronic stress in adulthood is unlikely to have any causal influence on chronological age, biological sex, or education. Conversely, stress and resilience could affect the included modifiable dementia risk factors through unhealthy coping behaviors like physical inactivity. Possible scores for the modified CAIDE dementia risk score range from 0 to 7, where higher scores reflect greater dementia risk.

### Other Variables

Other demographic variables, including age, sex, education, and race, were measured through self-report questionnaires. Race was dichotomized into White and non-White (African, Asian, Indigenous Australian, Latin American, Pacific Islander, or not specified) groups, given that the response for some categories was as small as 0.1% of the total sample.

### Statistical Analysis

All analyses were performed using R software, version 4.1.0 (R Core Team, 2019). Demographic data were calculated as the mean and standard deviation (for continuous variables) or the frequency and percentage (for categorical variables). We calculated Pearson correlation coefficients to determine the relationship between perceived stress, psychological resilience, cognition, and the modified dementia risk score. We then conducted separate multiple linear regression analyses for each predictor (perceived stress and psychological resilience). Outcomes were performance on the attention and working memory composites (as measured by the Cogstate Brief Battery), associative memory performance (as measured by the CANTAB PAL first attempt memory score), and the modified CAIDE dementia risk score. Age, sex, race, and years of education were included as covariates for all analyses. We did not include depressive symptoms as a covariate because these symptoms were strongly correlated with perceived stress (*r* = 0.7). Statistical significance was set at *p* < .05. We then conducted regression analyses to determine whether any association of perceived stress with cognition and the dementia risk score was modified by psychological resilience. Perceived stress and psychological resilience were first centered. The interaction term was computed by multiplying the two centered variables together. Statistical moderation was present if the interaction term between perceived stress and psychological resilience was statistically significant (*p* < .05). To adjust for multiple comparisons, the Benjamini–Hochberg procedure was used to control the false discovery rate (Benjamini & Hochberg, 1995).

Given the time interval between the completion of the CD-RISC questionnaire and cognitive testing, we sought to verify that this interval did not alter the results. Sensitivity analyses were conducted by repeating the primary analyses where psychological resilience was a predictor, including an additional adjustment for the time interval between the baseline CD-RISC and baseline cognitive outcomes.

## Results

Characteristics of the study sample are shown in Table 1. Overall, the sample was predominately White (82%) and female participants (76%). Participants were highly educated (*M* = 16 years) and most had a family history of dementia (65%). On average, participants scored highly on resilience (*M* = 29.7, out of a maximum possible score of 40), but low on perceived stress (*M* = 10.7, out of a maximum possible score of 40). Levels of perceived stress were moderately and inversely correlated with levels of psychological resilience (*r* = −0.48, *p* < .001) Correlations between other variables are shown in Table 2. See Supplementary Figure 1 for the distributions of the *z* scores across the three cognitive outcomes.

**Table 1.** Participant Characteristics

Participant characteristics	*N*	*M* ± *SD* [range]	*n* (%)
Age, years	2,266	57.1 ± 7.1 [40–71]	
Female	2,266		1,726 (76.2%)
Education, years	2,266	16.0 ± 3.5 [5–24]	
Race	2,266		
African			3 (0.1%)
Asian			52 (2.3%)
Indigenous Australian			26 (1.1%)
Latin American			7 (0.3%)
White			1,846 (81.5%)
Other			332 (14.7%)
Family history of dementia	2,249		1,477 (65.2%)
Modified CAIDE	1,913	1.3 ± 1.6 [0–7]	
Hypertension	1,913		365 (19.1%)
Hypercholesterolemia	1,913		353 (18.5%)
Physical activity	1,913		
Low			153 (8.0%)
Moderate			864 (45.2%)
High			896 (46.8%)
BMI, kg/m^2^	1,913	26.7 ± 5.7 [14.5–79.6]	
PSS, total score	2,266	10.7 ± 6.9 [0–40]	
CD-RISC, total score	2,266	29.7 ± 6.4 [0–40]	
PAL, total number of errors adjusted	1,522	12.9 ± 11.2 [0–59]	
PAL, first attempt memory score	1,522	13.2 ± 3.7 [2–20]	
Attention composite	1,709	0.0 ± 0.9 [−4.9 to 2.6]	
Working memory composite	1,709	0.0 ± 0.8 [−3.5 to 2.5]	

*Note*: BMI = body mass index; CAIDE = cardiovascular risk factors, aging, and incidence of dementia; CD-RISC = Connor–Davidson resilience scale; PAL = paired associates learning; PSS = perceived stress scale.

**Table 2.** Unadjusted Correlations Between Perceived Stress, Psychological Resilience, Cognition, and the Dementia Risk Score

	PSS	CD-RISC	Attention composite	Working memory composite	Associative memory	Modified CAIDE dementia risk score
PSS	—					
CD-RISC	−0.48**	—				
Attention composite	−0.04	0.10**	—			
Working memory composite	−0.09**	0.07*	0.07**	—		
Associative memory	−0.05	0.09*	0.16**	0.20**	—	
Modified CAIDE dementia risk score	0.10**	−0.09**	−0.10**	−0.07*	−0.04	—

*Notes*: CAIDE = cardiovascular risk factors, aging, and incidence of dementia; CD-RISC = Connor–Davidson resilience scale; PSS = perceived stress scale.

**p < .001; *p < .05.

### Associations of Perceived Stress and Psychological Resilience With Cognition

After controlling for age, sex, education, and race, perceived stress was significantly associated with all cognitive outcomes (Table 3). Specifically, higher levels of perceived stress were associated with poorer attention, working memory, and associative memory performances. Each 10-point increase in the PSS was associated with a 0.1 *z*-score unit decline in attention, working memory, and associative memory performance.

**Table 3.** Results of Separate Regression Analyses Evaluating the Association of Perceived Stress and Psychological Resilience with Cognitive Outcomes and Dementia Risk Score

	Attention composite (*n* = 1,709)		Working memory composite (*n* = 1,709)		Associative memory (*n* = 1,522)		Modified CAIDE dementia risk score (*n* = 1,913)	
Predictors	ß (*SE*)	*p* Value	ß (*SE*)	*p* Value	ß (*SE*)	*p*	ß (*SE*)	*p* Value
PSS	−0.010 (0.003)	**<.001**	−0.012 (0.003)	**<.001**	−0.013 (0.004)	**<.001**	0.031 (0.006)	**<.001**
CD-RISC	0.015 (0.003)	**<.001**	0.007 (0.003)	**.026**	0.014 (0.004)	**<.001**	−0.023 (0.006)	**<.001**

*Notes*: CAIDE = cardiovascular risk factors, aging, and incidence of dementia; CD-RISC = Connor–Davidson resilience scale; PSS = perceived stress scale.

Predictors (PSS and CD-RISC) were entered in separate models (i.e., the coefficient of PSS is not adjusted for CD-RISC and vice versa). All models adjusted for age, sex, race, and years of education. Beta coefficients are unstandardized. Bold typeface indicates significant result after correction for false discovery rate using the Benjamini–Hochberg procedure.

In separate linear regression models, psychological resilience was also significantly associated with all cognitive outcomes after controlling for age, sex, education, and race. Specifically, higher levels of resilience were associated with better attention, working memory, and associative memory performances (Table 3). For each 10-point increase on the CD-RISC, attention increased by 0.2 *z*-score units, and working memory and associative memory performance increased by 0.1 *z*-score units.

### Associations of Perceived Stress and Psychological Resilience With Dementia Risk Score

After adjusting for age, sex, years of education, and race, perceived stress was significantly associated with the modified CAIDE dementia risk score (Table 3). For every 10-point increase in the PSS, scores on the modified CAIDE dementia risk score increased by 0.3 original units. Additionally, psychological resilience was significantly associated with the modified CAIDE dementia risk score after adjusting for age, sex, years of education, and race (Table 3). For every 10-point increase on the CD-RISC, scores on the modified dementia risk score decreased by 0.2 original units.

### Moderation of Perceived Stress by Psychological Resilience

In moderation analyses, the interaction term between perceived stress and psychological resilience was added to regression models. After controlling for age, sex, education, and race, there was no evidence that psychological resilience moderated any associations of perceived stress with cognition or the modified CAIDE dementia risk score, as shown by the statistically nonsignificant (*p* > .05) interaction terms (see Supplementary Material).

### Sensitivity Analysis

We performed an additional analysis adjusting for the time between baseline CD-RISC data and baseline cognitive outcomes. Importantly, this did not substantially change the pattern of results (see Supplementary Material).

## Discussion

In this study, we examined the cross-sectional associations of perceived stress and psychological resilience with cognition and the burden of readily modifiable dementia risk factors in a middle-aged web-based sample without self-reported cognitive impairment. We found that higher levels of perceived stress and lower levels of psychological resilience were separately associated with poorer cognition, specifically in the domains of attention, working memory, and associative memory, although the magnitude of effect for these relationships was relatively small. Lower levels of perceived stress and higher levels of psychological resilience were also separately associated with lower dementia risk scores based only on modifiable risk factors. Overall, the results suggest that middle-aged individuals with lower levels of perceived stress or greater levels of psychological resilience have better cognition and a lower burden of modifiable dementia risk factors. However, we did not find evidence for an interaction between one’s levels of stress and psychological resilience when predicting cognition or modifiable dementia risk scores, suggesting that resilience does not buffer against the negative effects of stress.

The current results are consistent with previous studies that have demonstrated a significant association between higher levels of perceived stress and poorer cognition (Aggarwal et al., 2014; Chen et al., 2019; Oumohand et al., 2020). Although it has been suggested that memory (particularly memory tasks requiring a high cognitive load) is more vulnerable to the effects of perceived stress than other domains (VonDras et al., 2005), we found that attention, working memory, and associative memory performances were all negatively associated with perceived stress. These results suggest that systems involved in processing information are also sensitive to high levels of stress, not just those involved in the retention of information. Further research is needed to explore the factors that might mediate this association; for example, stress-related thoughts (i.e., worry or anxiety) might impair the efficiency of attention (Eysenck et al., 2007), which might then influence the performance on cognitive tasks that are heavily dependent on attention and working memory, such as those used in the current study.

We found that trait resilience was positively associated with performance on all cognitive domains measured. Although research investigating the relationship of psychological resilience with cognition in middle-aged or older adults remains limited, our findings are in accord with several studies (Deng et al., 2018; Jung et al., 2021; Ma et al., 2022; McDaniel et al., 2022). Importantly, we have extended previous work since, in addition to using a valid and standardized tool to assess psychological resilience, we also used cognitive measures that are more sensitive to the detection of cognitive deficits than cognitive screening tools, such as the Mini-Mental State Examination and Telephone Interview of Cognitive Status, used in previous studies (Jung et al., 2021; Lamond et al., 2008; McDaniel et al., 2022). Given the lack of research into the association between psychological resilience and cognition, the processes underlying this association are not well understood. However, results lend support to exploring psychological resilience as a potential protective factor for cognitive impairment.

Consistent with our hypothesis, higher levels of perceived stress and lower levels of psychological resilience were associated with a greater dementia risk score based on readily modifiable risk factors. The risk factors that comprise the modified CAIDE dementia risk score are primarily cardiovascular factors (e.g., blood pressure, body mass index, cholesterol, and physical activity). As such, our results support previous research highlighting links between stress and vascular health (Sara et al., 2022). In particular, chronic stress has been shown to be associated with hypertension (Liu et al., 2017), one modifiable factor included in the CAIDE dementia risk score. It is also postulated that poor coping strategies in response to stress, such as increased smoking, overeating, and physical inactivity (McDaniel et al., 2022; McEwen, 2008), might negatively affect vascular health. These findings support the hypothesis that the relationship of psychological stress and resilience with dementia risk could be mediated through vascular pathways.

It has previously been proposed that stress-coping ability might buffer against the negative effects of stress (Wilkinson et al., 2000). However, we did not find a significant interaction effect in the current study, suggesting that perceived stress has a negative association with cognition and dementia risk score, regardless of an individual’s level of trait resilience. In other words, high levels of trait resilience are associated with better outcomes at both low and high levels of perceived stress. Although these results suggest that interventions targeting both perceived stress and psychological resilience might be useful to improve cognition and lower dementia risk, given the cross-sectional nature of our study, longitudinal studies are needed to establish the temporal directionality of the observed associations. Though the association between perceived stress and cognitive decline over time has been investigated (Aggarwal et al., 2014; Chen et al., 2019; Turner et al., 2017), research investigating the association between psychological resilience and cognitive decline is needed.

A strength of the current study was the large community-based sample of middle-aged adults. One advantage of a middle-aged sample is a lower probability that any observed associations of stress or resilience with cognition and dementia risk scores are influenced by incipient dementia or substantial underlying neurodegenerative pathology. However, it is also important to investigate the observed associations in samples of older adults, to determine if the associations observed in the current study will generalize to older adults, as modifying risk factors in late life may still ameliorate dementia risk (Livingston et al., 2020). A second strength is our use of a validated measure of psychological resilience. Several previous studies have chosen to define psychological resilience as individuals who remain “mentally healthy” regardless of their experience of adverse events (e.g., no depressive symptoms in the presence of at least one negative life event; Jung et al., 2021). Given that depression is associated with cognitive impairment and dementia risk (Butters et al., 2008; Livingston et al., 2020), including depressive symptoms in the measurement of resilience could obscure any relationship between resilience and cognition.

Several limitations warrant consideration. First, the observational and cross-sectional design prevents conclusions regarding causality. Second, given the exclusion of participants with psychiatric disorders in the past year and current untreated depression, it is possible that a large proportion of individuals who are likely to experience stress might be excluded. Third, the generalizability of our findings to other populations may be limited given the large proportion of White highly educated females with a family history of dementia included in the current study. Last, given the CD-RISC was added to the HBP platform after the study commencement, there was a delay between completion of the CD-RISC and baseline cognitive outcomes. We attempted to address this delay by conducting a sensitivity analysis, which showed that additional adjustment for the time interval between the CD-RISC and cognitive measures did not significantly change the findings. This may in part be because psychological resilience is relatively stable over time (Connor & Davidson, 2003). Nonetheless, it would be useful for future studies to replicate these results when measuring resilience at the same time point as perceived stress.

In conclusion, we identified that perceived stress and psychological resilience were separately associated with cognitive performance and the burden of modifiable dementia risk factors. However, there was no interaction between perceived stress and psychological resilience, suggesting that perceived stress is associated with cognition and modifiable dementia risk factors, regardless of one’s levels of psychological resilience. To translate our findings, intervention studies are required to determine if lowering stress and building resilience can mitigate cognitive deficits and reduce dementia risk scores, as targeting modifiable risk factors is a promising means to reduce dementia prevalence.

## Supplementary Material

gbad131_suppl_Supplementary_Material
