# Supplementary material for: Associations of Perceived Stress and Psychological Resilience With Cognition and a Modifiable Dementia Risk Score in Middle-Aged Adults
Source: J Gerontol B Psychol Sci Soc Sci. 2023 Sep 18;78(12):1992–2000. doi: 10.1093/geronb/gbad131 (PMC10699744; doi:10.1093/geronb/gbad131)
Supplement: gbad131_suppl_Supplementary_Material [file gbad131_suppl_supplementary_material.docx]

**Supplemental material for:** Associations of perceived stress and psychological resilience with cognition and a modifiable dementia risk score in middle-aged adults.

Franks, K. H., Bransby, L., Cribb, L., Buckley, R., Yassi, N., Chong, T. T.-J., Saling, M. M., Lim, Y. Y., & Pase, M. P.

**Supplementary Figure 1. Distribution of z-scores on each cognitive outcome.**

**Supplementary Figure 2. Interaction plots displaying the non-significant moderation effect of psychological resilience on the relationship of perceived stress with cognition and the modified dementia risk score.**

**Supplementary Table 1. Calculation of the modified CAIDE dementia risk score.**

**Supplementary Table 2. Results of separate regression analyses evaluating the association of perceived stress with cognitive outcomes and dementia risk score.**

**Supplementary Table 3. Results of separate regression analyses evaluating the association of psychological resilience with cognitive outcomes and dementia risk score.**

**Supplementary Table 4. Results of regression analyses including the interaction between perceived stress and psychological resilience on cognitive outcomes and dementia risk score.**

**Supplementary Table 5. Results of sensitivity analyses with additional adjustment for the time interval between baseline CD-RISC and cognitive outcomes.**

**Supplementary Figure 1.** Distribution of z-scores on each cognitive outcome.


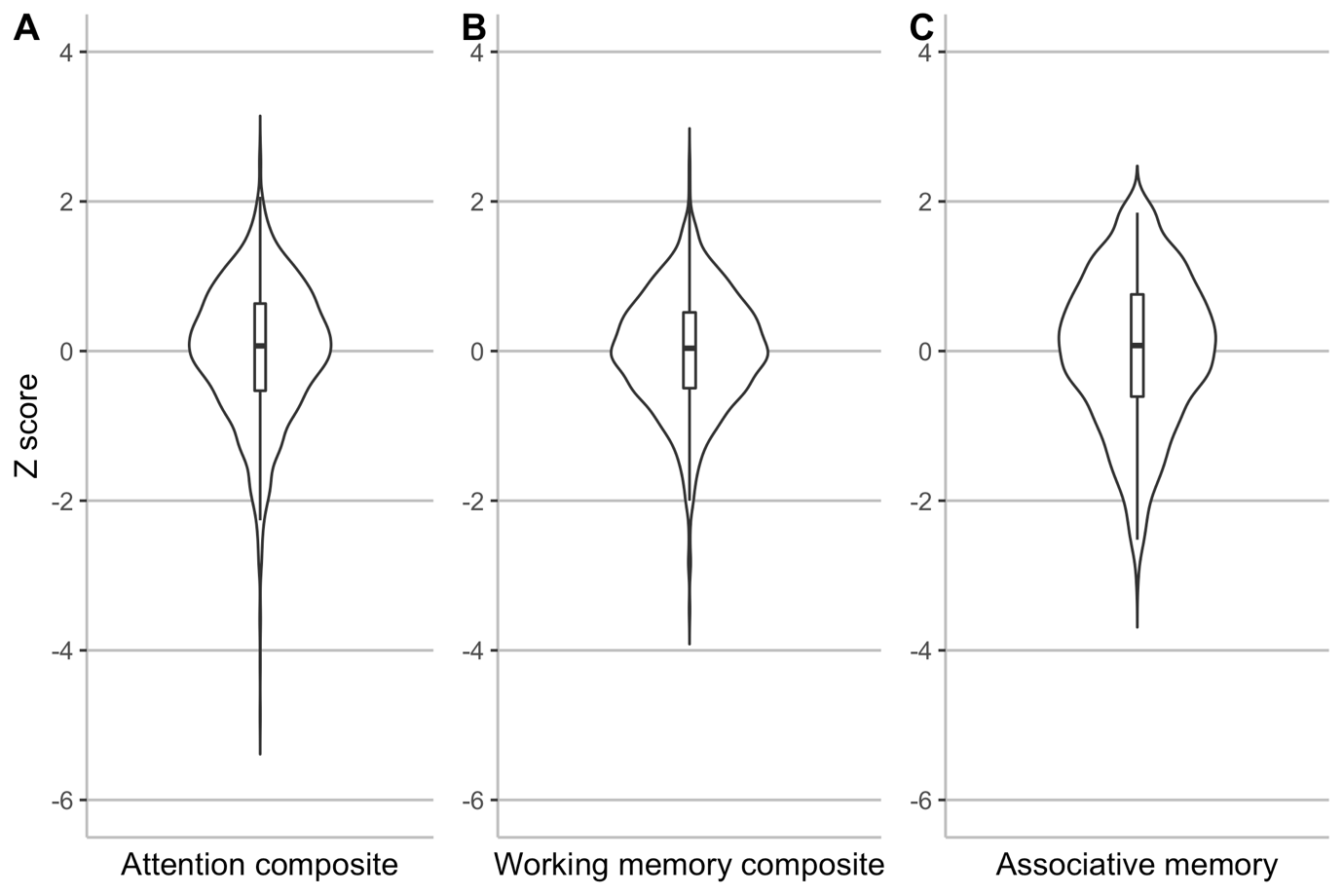


**Supplementary Figure 2.** Interaction plots displaying the non-significant moderation effect of psychological resilience on the relationship of perceived stress with cognition and the modified dementia risk score.


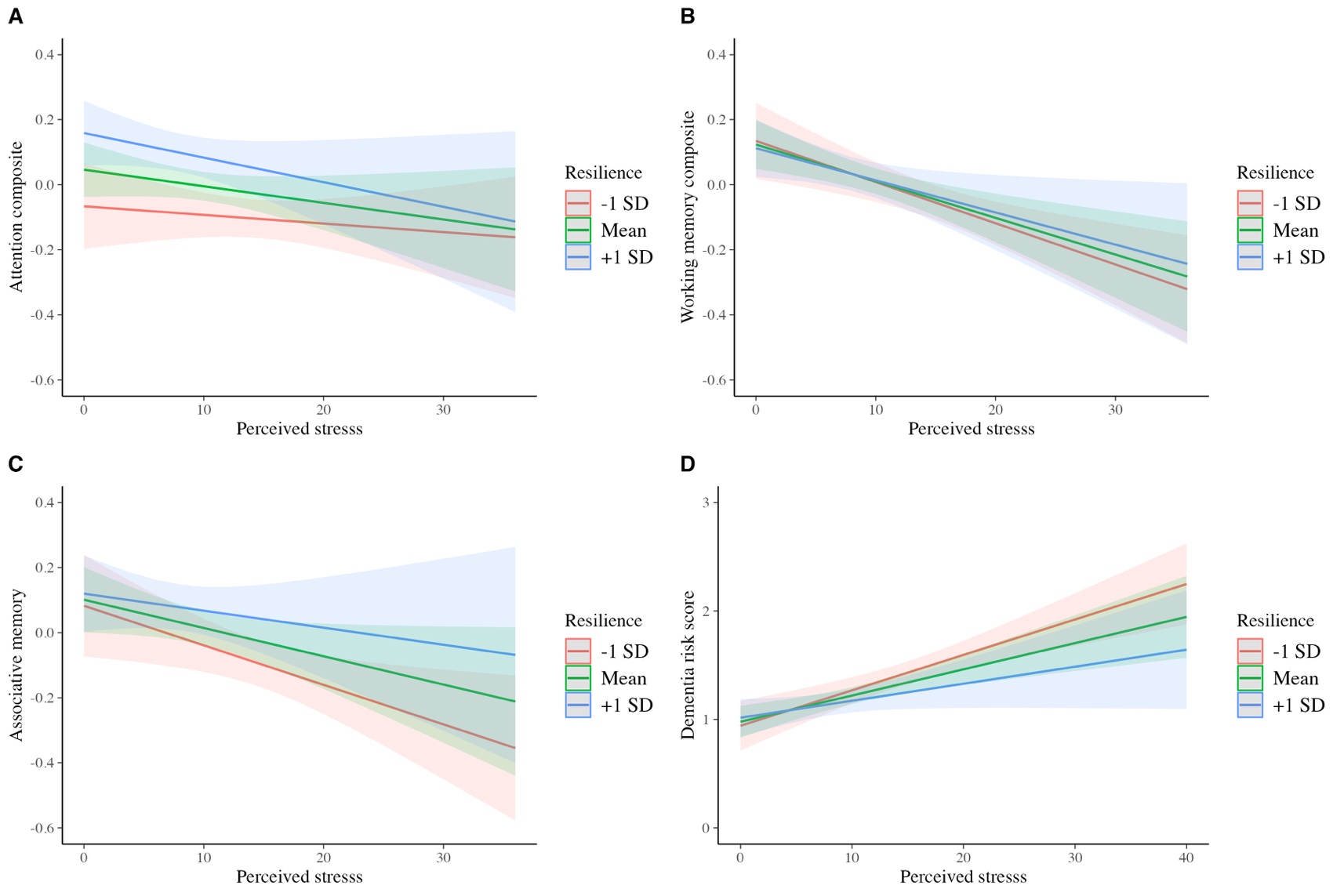


**Supplementary Table 1.** Calculation of the modified CAIDE dementia risk score.

| Factors | Original CAIDE (points) | Modified CAIDE (points) | Measurement |
| --- | --- | --- | --- |
| Age |  | - |  |
| <47 years | 0 |  | Calculated from self-reported date of birth. |
| 47-53 years | 3 |  |  |
| >53 years | 4 |  |  |
| Sex |  | - |  |
| Female | 0 |  | Self-reported |
| Male | 1 |  |  |
| Education |  | - |  |
| ≥10 years | 0 |  | Self-reported |
| 7-9 years | 2 |  |  |
| 0-6 years | 3 |  |  |
| Systolic blood pressure |  |  |  |
| ≤140 mmHg | 0 | 0 | Self-reported history of hypertension (yes/no). |
| >140 mmHg | 2 | 2 |  |
| BMI |  |  |  |
| ≤30 kg/m^2^ | 0 | 0 | Calculated from self-reported height and weight. |
| >30 kg/m^2^ | 2 | 2 |  |
| Cholesterol |  |  |  |
| ≤6.5 mmol/L | 0 | 0 | Self-reported diagnosis of high cholesterol (without diagnosis scored as 0, with diagnosis scored as 2). |
| >6.5 mmol/L | 2 | 2 |  |
| Physical activity |  |  |  |
| Active | 0 | 0 | Self-reported physical activity was assessed with the IPAQ (“High” or “Moderate” activity levels on the IPAQ were scored as 0, while “Low” activity levels were scored as 1). |
| Inactive | 1 | 1 |  |
|  | Maximum 15 points | Maximum 7 points |  |

BMI, body mass index; CAIDE, Cardiovascular Risk Factors, Aging, and Dementia; IPAQ, International Physical Activity Questionnaire.

**Supplementary Table 2.** Results of separate regression analyses evaluating the association of perceived stress with cognitive outcomes and dementia risk score.

|  | Attention composite  (*n* = 1709) | | Working memory composite  (*n* = 1709) | | Associative memory  (*n* = 1522) | | Modified CAIDE dementia risk score  (*n* = 1913) | |
| --- | --- | --- | --- | --- | --- | --- | --- | --- |
| *Predictors* | *ß* (SE) | *p* | *ß* (SE) | *p* | *ß* (SE) | *p* | *ß* (SE) | *p* |
| PSS | -0.010 (0.003) | **<.001** | -0.012 (0.003) | **<.001** | -0.013 (0.004) | **<.001** | 0.031 (0.006) | **<.001** |
| Age | -0.038 (0.003) | **<.001** | -0.006 (0.003) | **.024** | -0.032 (0.004) | **<.001** | 0.034 (0.005) | **<.001** |
| Sex | -0.016 (0.049) | .740 | -0.055 (0.044) | .209 | -0.096 (0.060) | .107 | 0.209 (0.086) | **.015** |
| Education | 0.015 (0.006) | **.018** | 0.024 (0.005) | **<.001** | 0.021 (0.007) | **.005** | -0.036 (0.011) | **<.001** |
| Race | 0.052 (0.055) | .342 | 0.048 (0.049) | .328 | -0.014 (0.004) | **<.001** | -0.002 (0.095) | .982 |

All models adjusted for age, sex, years of education, and race. Beta coefficients are unstandardised. Bold typeface indicates significant result.

*Note.* CAIDE, Cardiovascular Risk Factors, Aging, and Incidence of Dementia; CD-RISC, Connor-Davidson Resilience Scale; PSS, Perceived Stress Scale

**Supplementary Table 3.** Results of separate regression analyses evaluating the association of psychological resilience with cognitive outcomes and dementia risk score.

|  | Attention composite  (*n* = 1709) | | Working memory composite  (*n* = 1709) | | Associative memory  (*n* = 1522) | | Modified CAIDE dementia risk score  (*n* = 1913) | |
| --- | --- | --- | --- | --- | --- | --- | --- | --- |
| *Predictors* | *ß* (SE) | *p* | *ß* (SE) | *p* | *ß* (SE) | *p* | *ß* (SE) | *p* |
| CD-RISC | 0.015 (0.003) | **<.001** | 0.007 (0.003) | **.026** | 0.014 (0.004) | **<.001** | -0.023 (0.006) | **<.001** |
| Age | -0.037 (0.003) | **<.001** | -0.005 (0.003) | .091 | -0.030 (0.004) | **<.001** | 0.030 (0.005) | **<.001** |
| Sex | -0.010 (0.049) | .845 | -0.050 (0.044) | .258 | -0.092 (0.060) | .121 | 0.195 (0.086) | **.024** |
| Education | 0.012 (0.006) | .066 | 0.024 (0.006) | **<.001** | 0.018 (0.008) | **.018** | -0.033 (0.011) | **.003** |
| Race | 0.063 (0.055) | .247 | 0.058 (0.049) | .233 | 0.137 (0.066) | **.039** | -0.021 (0.095) | .826 |

All models adjusted for age, sex, years of education, and race. Beta coefficients are unstandardised. Bold typeface indicates significant result.

*Note.* CAIDE, Cardiovascular Risk Factors, Aging, and Incidence of Dementia; CD-RISC, Connor-Davidson Resilience Scale; PSS, Perceived Stress Scale

**Supplementary Table 4.** Results of regression analyses including the interaction between perceived stress and psychological resilience on cognitive outcomes and dementia risk score.

|  | Attention composite  (*n* = 1709) | | Working memory composite  (*n* = 1709) | | Associative memory  (*n* = 1522) | | Modified CAIDE dementia risk score  (*n* = 1913) | |
| --- | --- | --- | --- | --- | --- | --- | --- | --- |
| *Predictors* | *ß* (SE) | *p* | *ß* (SE) | *p* | *ß* (SE) | *p* | *ß* (SE) | *p* |
| PSS | -0.034 (0.024) | .161 | -0.075 (0.022) | **<.001** | -0.058 (0.029) | .046 | 0.164 (0.043) | **<.001** |
| CD-RISC | 0.087 (0.024) | **<.001** | 0.003 (0.021) | .879 | 0.055 (0.029) | .059 | -0.053 (0.042) | .211 |
| PSS X CD-RISC | -0.016 (0.019) | .381 | 0.009 (0.017) | .576 | 0.023 (0.022) | .297 | -0.058 (0.033) | .077 |

All models adjusted for age, sex, race, and education. Beta coefficients are unstandardised. Estimates taken from final step of the model. Bold typeface indicates significant result after correction for false discovery rate using the Benjamini-Hochberg procedure.

*Note*. CAIDE, Cardiovascular Risk Factors, Aging, and Incidence of Dementia; CD-RISC, Connor-Davidson Resilience Scale; PSS, Perceived Stress Scale

**Supplementary Table 5**. Results of sensitivity analyses with additional adjustment for the time interval between baseline CD-RISC data and cognitive outcomes.

|  | Attention composite  (*n* = 1709) | | Working memory composite  (*n* = 1709) | | Associative memory  (*n* = 1522) | |
| --- | --- | --- | --- | --- | --- | --- |
| *Predictor* | *ß* (SE) | *p* | *ß* (SE) | *p* | *ß* (SE) | *p* |
| CD-RISC | 0.015 (0.003) | **<.001** | 0.006 (0.003) | **.029** | 0.014 (0.004) | **<.001** |

Note: models adjusted for age, sex, race, education, and time interval between baseline CD-RISC and cognitive measures. Beta coefficients are unstandardised. Bold typeface indicates significant result after correction for false discovery rate using the Benjamini-Hochberg procedure.

*Note.* CD-RISC, Connor-Davidson Resilience Scale
